# Evaluation of the medical student research programme in Norwegian medical schools. A survey of students and supervisors

**DOI:** 10.1186/1472-6920-9-43

**Published:** 2009-07-14

**Authors:** Steinar Hunskaar, Jarle Breivik, Maje Siebke, Karin Tømmerås, Kristian Figenschau, John-Bjarne Hansen

**Affiliations:** 1 Faculty of Medicine and Dentistry, University of Bergen, Bergen, Norway; 2 Faculty of Medicine, University of Oslo, Oslo, Norway; 3 Faculty of Medicine, Norwegian University of Science and Technology, Trondheim, Norway; 4 Faculty of Medicine, University of Tromsø, Tromsø, Norway

## Abstract

**Background:**

The Medical Student Research Programme is a national education and grant scheme for medical students who wish to carry out research in parallel with their studies. The purpose of the programme is to increase recruitment of people with a standard medical degree to medical research. The Research Programme was established in 2002 and underwent a thorough evaluation during the spring of 2007. The evaluation should investigate if the programme had fulfilled its objectives of increased recruitment to medical research, in addition to the students' and supervisors' satisfaction of the programme, and unwanted differences between the universities.

**Methods:**

Data was collected from students, supervisors and administrative staff via web-based questionnaires. Information about admission, implementation, results achieved and satisfaction was analysed and compared between the four Norwegian medical schools. In addition, the position of the scheme in relation to the national Quality Reform of Higher Education was analysed.

**Results:**

At the end of 2006, the Medical Student Research Programme had recruited 265 medical students to research. These consisted of 214 active students, 35 who had completed their studies and only 17 who had dropped out. Both students and supervisors were generally very satisfied with the scheme, including the curriculum, the results achieved and the administrative service. The majority of students wanted to continue their research towards a PhD and, of those who had completed the Medical Student Research Programme, practically all had published one or several scientific papers. The survey showed only small differences between the four medical schools, despite their choice of somewhat different solutions in terms of administration and organisation. The Medical Student Research Programme satisfies the majority of the demands of the Quality Reform, however as an integrated research programme aimed at a PhD it presupposes access to PhD courses before the completion of medical studies, as well as the ability to include undergraduate scientific work in a PhD thesis.

**Conclusion:**

The Medical Student Research Programme has led to an increase in the recruitment of graduated physicians to medical research in Norway. It will only be possible to evaluate whether this in turn will result in a larger number of PhDs in 3–5 years; this will also depend on the access to grants and fellowships.

## Background

In the late 1990s, the recruitment of medical graduates to medical research in Norway was worryingly low. The number of doctors choosing research as a career had fallen, and the fear was that this might have serious consequences both for the health service and for the universities. It had already become difficult to identify qualified applicants to many medical professorships. The figures also showed a dramatic reduction in the number of Norwegian medical students recruited to student research fellowships [1]. During the period 1990–97, the number of funded research students financed by the Research Council of Norway and the Norwegian Cancer Society fell by 80% in relation to the increase in the number of study places. In 1997, funded research students made up a mere 2% of all medical students, despite the positive evaluation of the scheme, the large number of funded research students who completed PhDs and the fact that they did so at a younger age than other doctors [2].

The establishment of the Medical Student Research Programme was agreed by the Norwegian Parliament in 2001, and the scheme was implemented in all the four medical faculties in Norway (Bergen, Oslo, Tromsø, and Trondheim). The scheme is based on the earlier scheme for funded student research at the Research Council of Norway, while responsibility for implementation and quality assurance lies with each medical school. The research positions are still funded via a framework grant from the Research Council, while the running of the Programme is financed by a central government grant as well as from incentive-based funds based on the generation of credit points. The Research Council grant is calculated from the intention that the Medical Student Research Programme should be offered to up to 10% of medical students.

In the spring of 2006, the four research deans established a working group in order to evaluate the first 5 years of the Medical Students Research Programme. The purpose of the evaluation was to investigate if the programme had fulfilled its objectives of increased recruitment to medical research, the students' and supervisors' satisfaction of the programme, and unwanted differences between the universities. If possible, success factors and problems should be identified. The evaluation has consisted of a systematic review of the history of the Research Programme, its organisation and relationship to the Quality Reform, as well as a web-based user survey aimed at students, supervisors and administrative staff.

## Methods

The main nationally common elements of the Medical Student Research Programme are shown in Table 1. However, the organisation of the scheme varies somewhat between the four medical schools [3-6], including different admission times, different courses and some variation in the allocation of funding. Table 2 shows more details of the educational and scientific part of the program, and also some of the differences between the universities regarding staff and funding. The Medical Student Research Programmes are organised under the Vice Dean for research at the individual schools, and managed by one faculty member (professor or postdoctoral level) and one administrative leader whose work is closely associated with the faculty, the medical school, the PhD programme administration and the research environments. The management is responsible for the academic aspects of the study, and actively recruit and follow up students and supervisors.

**Table 1:** Common elements of the Medical Student Research Programmes at the Norwegian medical schools

**Element**	**Description**
Length of curriculum	All Norwegian medical schools have a six year curriculum. The Medical Student Research Programme is a two-year course (120 credit points), one of these years is added to the study of medicine and one year is integrated into the curriculum as extra work time and scientific work during weekends and summers. The total duration of the study of medicine with a Research Programme thus becomes 7 years
Recruitment	Up to 10% of the year groups in each medical school can be recruited annually. There are currently 20 study places at UiO, 15 at UiB, 12 at NTNU and 6 at UiT annually
Formalised research training	30 credit points, which equals an ordinary PhD programme
Research and publications	Research and the research paper/publication(s) give 90 credit points
Elements of curriculum	The course consists of full-time research for one year (this could be two separate terms), research courses and seminars, summer schools and research work in parallel with ordinary medical school
Assessment	The course is assessed with pass/no pass
Certificate and approval	The formalised research training may be approved as the theoretical part of the PhD-programme
Postgraduate training	The course implies shorter specialist training and further studies leading up to a PhD. A completed Research Programme counts as one year's research in specialist training for all specialist fields
Funding of students	The Research Council of Norway funds a 2-year grant per student. The annual grant for 2008 is NOK 96,000 (USD 15,000, EUR 12,000)

**Table 2:** Details of the educational and scientific part of the program, scientific and economic support, by the four universities (2007)

	**NTNU**	**UiB**	**UiO**	**UiT**
**Human resources**				
Scientific leader	Postdoctor, 50%	Professor, 100%	Professor, 100%	Professor, 50%
Administrative staff	50%	50%	100%	50%
**Programme management**				
Programme start (months of medical school)	24	18 or later	24 or later	6–36
Progress report	Annually	Annually	Annually	Annually
Minimum scientific requirement for pass (exam procedure)	Monography or thesis with description of project + 1–2 original papers published. Student as first or second author. Oral presentation and defence for evaluation committee	Original paper published or in press. Student as first or second author. Evaluation by programme leader. If no publication; monography + declaration from student and supervisor	Review of minimum 5000 words + original paper published or in press. Student as first or second author. Oral presentation and defence for evaluation committee	Published paper or in press. Student as first or second author. Oral presentation and defence for evaluation committee
**Compensation for running costs**				
To student directly	0	NOK 50,000, USD 8,000, EUR 6,000	0	For travel. NOK 20,000, USD 3,000, EUR 2,500
To supervisor	NOK 70,000, USD 11,000, EUR 9,000	NOK 30,000, USD 5,000, EUR 4,000	NOK 205,000 (lab research), 100,000 (else), USD 32,000/16,000, EUR 25,000/12,000	NOK 200,000 (lab research), 100,000 (else), USD 31,000/16,000, EUR 25,000/12,000
To department	0	0	NOK 30,000, USD 5,000, EUR 4,000	0
**Educational part**				
Formal PhD programme (30 credits)	Yes	Yes	Yes	Yes
Mandatory part	8 credits	8 credits	23 credits	13 credits
Mandatory laboratory animal research course	Yes, if relevant to project	Yes, if relevant to project	Yes, if relevant to project	Yes, if relevant to project
Courses given by the programme itself	0 credits	1 credit	23 credits	8 credits

### The survey

The working group established by the four deans in order to carry out a national evaluation consisted of 10 members, including representatives of the administrative and technical management of the medical schools as well as representatives of students and supervisors from the Research Programme. The mandate of the working group was to suggest content, technical solutions and method for interpreting the data in connection with the evaluation of the Programme. It was decided to create a web-based registration form for former and current students, supervisors and administrators on the Research Programmes (same structure, but edited specifically for each of the groups). The programme Quest-back was used.

The task of the questionnaire was to reveal the participants' satisfaction with specific and organisational aspects of the Research Programmes, and to what extent they had reached the goal of increased recruitment of students to medical research. The questionnaire for current and former students consisted of the following parts:

- demographic variables and identification of university-time of enrolment

- classification of project

- volume and frequency of supervision and satisfaction with the supervisor

- personal aims for the research

- scientific output in form of publications and manuscripts, presentations of research results at national/international conferences, or field studies abroad-satisfaction with services from the local Research Programme

- ambitions of and recruitment to subsequent PhD projects-interference with the ordinary curriculum

- global satisfaction with the programme

The questionnaire for supervisors consisted of the following parts:

- demographic variables, identification of professional level and name of university

- classification of main research interest (basic, laboratory, clinical, public health/epidemiology)

- information about the research group (size, kind of persons, scientific level, number of PhD students)

- volume and frequency of supervision-personal aims for the student

- evaluation of the student's level of progress, interest and ambitions for future research-satisfaction with services from the local Research Programme

- global satisfaction with the programme

The questionnaire for administrative personnel was used to collect information about the curricula and study programs, enrolment and dropouts, and other administrative information.

The questionnaires consisted mainly of quantitative and categorical answering options, but also some open questions with the opportunity to comment and give more extensive replies. Questionnaires were only sent to main supervisors. Some supervise more than one student on the Research Programme, and in such cases one form should be completed for each student. Supervisors count as one for each Research Programme student, and not as a number of different people. Before the questionnaire was sent out, a pilot registration was carried out among three Research Programme students and three supervisors at the University of Tromsø. The questionnaires were sent out on 15 January 2007 and the deadline was 1 February 2007. The survey was not anonymous, but all replies were sent to the University of Tromsø where the data files were made non-anonymous before statistical analyses and distribution to the individual universities.

An evaluation like this one is not subject to formal approval by the Regional Committee for Medical Research Ethics, Western Norway (REC West) according to Norwegian regulations in force, and the study was therefore not presented for the REC in advance. Upon later request, REC West has stated that this judgement was correct and that it has no objection to publication of the study.

We are presenting data only from the quantitative part of the survey as very few respondents answered the open questions. The data are mainly treated descriptively for the whole student population and supervisors, regardless of their institution. However, statistical analyses have been carried out in order to reveal any significant differences in the responses between the universities. One-way ANOVA is used for continual variables, while a chi square test is used for categorical variables. A two-way p-value < 0.05 was defined as statistically significant. SPSS version 14.0 was used for the analyses.

## Results

### The students

Questionnaires were sent to 208 active students, and 183 (87%) replied. Table 3 shows characteristics of the students on the Research Programme who responded to the survey, distributed throughout the medical schools. There was a significant difference between the faculties as regards how far the students had come in their ordinary studies when they were admitted to the Research Programme (p < 0.001). The most prominent difference between the faculties was that several students at the University of Oslo were admitted at a later stage in their studies. As far as classification of the research projects is concerned, approximately 60% have indicated that they carry out laboratory research. At the University of Bergen, more students classified their Research Programme projects as public health/epidemiology than any of the other universities.

**Table 3:** Characteristics of the Research Programme students who participated in the survey

	**NTNU**	**UiB**	**UiO**	**UiT**	**Total**
Number of students receiving questionnaire	53	70	67	18	208
Responses (%)	46 (87)	58 (83)	61 (91)	18 (100)	183 (87)
Age (years) (range)	24 (20–32)	25 (20–33)	25 (21–39)	24 (21–27)	25 (20–39)
Sex (% women)	35	52	43	39	43
Completed terms on admission. N and (%)					
1–2 terms	6 (13)	23 (40)	5 (8)	11 (61)	46 (25)
3–4 terms	37 (80)	35 (60)	25 (41)	6 (33)	102 (56)
5+ terms	3 (7)	0 (0)	31 (51)	1 (6)	35 (19)
Completed terms at time of response. N and (%)					
<2 terms	13 (29)	12 (21)	14 (23)	1 (6)	40 (22)
2–3 terms	12 (27)	13 (22)	13 (21)	5 (28)	44 (24)
4–5 terms	9 (20)	19 (33)	20 (33)	4 (22)	51 (28)
≥ 6 terms	11 (24)	14 (24)	14 (23)	8 (44)	48 (26)
Classification of projects. N and (%)					
Public health/epidemiology	1 (2)	12 (21)	6 (10)	2 (11)	22 (12)
Clinical/patient-oriented	10 (22)	15 (26)	11 (18)	3 (17)	38 (21)
Laboratory research	28 (61)	26 (45)	41 (67)	11 (61)	106 (58)
Other	7 (15)	5 (9)	3 (5)	2 (11)	17 (9)

The Research Programme students were very (46%) or somewhat (38%) satisfied with the Programme. As many as 47% felt that the Medical Student Research Programme was greatly or very useful to ordinary medical studies, whilst only 8% felt that it was not useful. Three-quarters of the students were satisfied with the supervision, while 19% said they required more supervision. The students felt that their supervisors had very high (7%) or high (63%) expectations of them, and practically all felt that the supervisors' expectations of them were well in keeping with their own. Only eight students indicated that they were dissatisfied with the research group they belonged to. The vast majority (80%) of the Research Programme students wanted to do a PhD, most of them (69%) in connection with the project they were involved in, 18% were unsure, while only 2% indicated that they had no such ambitions. There were no statistically significant differences in the distribution of replies from the students of the various faculties for any of the above topics.

Half of the current students had published or were about to submit scientific articles. Based on given lists of references from current and previous students we found a total of 61 publications, 18 publications from the Norwegian University of Science and Technology, 18 from the University of Bergen, 23 from the University of Oslo and 2 publications from the University of Tromsø. Further, 42% of the students said that they had had carried out one or several presentations (orally or by poster) at national conferences during the past year, and 27% had had one or several international presentations. One in seven of the students had carried out scientific field studies of minimum two weeks' duration abroad. There were no statistically significant differences in the distribution of replies from the students of the various faculties.

### Research Programme graduates

A total of 22 (63%) of the 35 students who graduated from the Research Programme before the end of 2006 responded to the survey. The majority (86%) characterised their projects as laboratory-based. Everyone, except one, reported having published scientific or popular science articles during their research studies. The vast majority (82%) was a little, or very satisfied with the Medical Student Research Programme. One in three had formally embarked on a PhD project, while 55% replied that they had ambitions in this respect. Nobody replied that they had no ambitions of a doctorate. One student of the Norwegian University of Science and Technology had already obtained a PhD. Most of the students had started their PhD immediately on completion of the Research Programme (50%) or during the following two terms (40%).

### The supervisors

71% of the 208 supervisors responded, the University of Oslo had the highest percentage of replies (88%) and the Norwegian University of Science and Technology the lowest (55%). The average age of the supervisors was 51 years, 25% were women and the majority were professors (76%) with their main position connected to the universities (74%). When asked how many hours of supervision they had given the students on the Research Programme during the past year, 80% said less than 40 hours, while 9% said more than 80 hours. This corresponded well with information given by the students. Nearly all (93%) of the supervisors experienced that students to some or to a great extent were a resource in the research team, and 66% thought that they would probably continue the supervision relationship after having completed the Medical Student Research Programme.

### Research Programme administration

Characteristics of student admission, implementation and student satisfaction with the administrative service are shown in Table 4. With the exception of the University of Bergen there were more applicants than study places on the Medical Student Research Programme in 2006. Six students (2%) dropped out in 2006 without graduating. The majority was satisfied with the service from the administration, while 11% was dissatisfied. Fewer students were satisfied with the administration of the Medical Student Research Programme at the University of Bergen and the Norwegian University of Science and Technology compared to the University of Oslo and the University of Tromsø (p < 0.001). Nine out of ten students experienced that they received satisfactory answers to questions and requests, but significantly more students at the University of Tromsø experienced access to information on the website as low or very low (61%) compared with the other faculties (9%) (p < 0.001).

**Table 4:** Characteristics of student admission, implementation and student satisfaction with the administrative service of the Medical Student Research Programme

	**NTNU**	**UiB**	**UiO**	**UiT**	**Total**
Annual number of medical students	120	150	210	100	580
Places on Medical Student Research Programme per year	12	15	20	6	53
Number of applicants in 2006	18	9	22	8	57
Total admissions 2002–2006	58	80	99	28	265
Number of students on 31.12.06	53	70	67	24	214
Total number of students opting out (% of all)	0 (0)	5 (6)	8 (8)	4 (14)	17 (6)
Number of students leaving during the past year (% of all)	0 (0)	3 (4)	1 (1)	2 (7)	6 (2)
Number of degrees awarded	4	5	26	0	35
Administrative service. N and (%)					
Very satisfied	5 (11)	21 (36)	35 (57)	5 (28)	66 (36)
Somewhat satisfied	19 (41)	10 (17)	16 (26)	8 (44)	53 (29)
Neither satisfied nor dissatisfied	15 (33)	18 (31)	9 (15)	2 (11)	44 (24)
Somewhat dissatisfied	7 (15)	7 (12)	1 (2)	1 (6)	16 (9)
Very dissatisfied	0 (0)	2 (3)	0 (0)	2 (11)	4 (2)

## Discussion

The Medical Student Research Programmes have probably more than doubled the number of Norwegian medical students who are carrying out research in parallel with their studies. Now it is approximately 10% of students per year, compared with about 2–4% engaged in research 10 years ago. The Research Programme scores highly, both among students and supervisors and has vitalised interest in research among students. The survey indicates that the Research Programme provides increased recruitment of well-qualified PhD candidates; however this overriding aim can only be evaluated in 3–5 years.

### Strengths and limitations of the study

The response rate is very satisfactory, and the survey must be presumed to give a valid picture of the situation. As the survey was not anonymous, bias based on that cannot be excluded, however. Although the number of research students is reached as aimed at each university, the total numbers for statistical analyses are low for some analyses, thus giving the calculations somewhat low statistical power. However, we base our interpretations and comments mainly on the overall descriptive pattern of the answers.

### Increased recruitment to medical research

The Medical Student Research Programmes at the four medical faculties in Norway were established in 2002 because of a worrying reduction of the number of medical students for student research fellowships and a fall in the proportion of PhD students with a standard medical degree entering medical research during the 1990s. An analysis of the resources going into medical and health-related research in 2005 showed a further reduction of the proportion of graduate physicians among the PhD students at universities after 1997 [7]. The proportion fell from 53% in 1997 to 35% in 2005, and the reduction was clear in all medical fields. From 2008, approximately 50 candidates will graduate from the Medical Student Research Programme every year, and this survey shows that at least 80% of the students have ambitions about obtaining a doctorate. Consequently there will be a need for between 40 and 45 research fellow positions for graduates from the Research Programme already as early as 2008–2009. It will have to be decided whether the Research Programme graduates have to compete for already established positions, or whether shorter PhD programmes for Research Programme graduates are to be established. As the full effect on the number of annual doctorates will not be seen until 2010–12, it is a bit early to be sure of the benefits, but an interim analysis like the present was seen necessary by the Deans.

Unlike the previous student research grant scheme, the Medical Student Research Programme is organised at the actual medical faculties with their own administrative services with technical and administrative local resources. Their task is recruitment, structured education of researchers, follow-up of the supervisor-student relationship and preparation of arenas for presentations and feedback about the students' presentations. All this may contribute to the high degree of completion. The survey shows a generally high level of satisfaction among the students, previous students and supervisors. Our findings indicate that students from the Research Programme, to a greater extent than was the case for previous student researchers, will complete a doctorate. It has been shown also in previous studies that an early experience of research stimulates an interest in further research, and ambitions about obtaining a PhD [8]. The full effect of the Medical Student Research Programme will therefore be totally dependent on a sufficient number of research fellowships.

The Medical Students Research Programme is a national initiative to increase the recruitment, and thus the proportion, of researchers with a standard medical degree within all medical fields. The results of the survey show that all the main fields of medical research have been included in the new population of fresh researchers; in fact the spread is probably better than before. In addition, the recruitment of researchers at student level has several advantages compared to the recruitment of graduate medical doctors. The students are already in an established education situation where motivation, financing and family relationships are favourable to research. The financial aspect in particular has been emphasised as a factor which limits the recruitment of doctors into research. The costs of grants to Medical Students Research Programme are considerably lower than for ordinary grants, at the same time as the students get full grant cover and do not have to take out additional student loans. Socio-economically it also makes sense to educate doctors to become competent researchers at an early stage as their professional careers will be that much longer. The Medical Students Research Programmes also carry out active recruitment work which may be used both in the faculties' own and in the national research strategy – both in terms of strengthening existing research environments and of developing new areas of research.

### Same overall result despite different schedules between the universities?

As shown in Table 2 there are several significant differences between the ways the programmes are organized at the four universities. Despite these differences in human resources, programme management, compensations for running costs during projects, and courses, there seems to be an overall fulfilment of the objectives of the programme across universities. This indicates that local management secures the result dependent of local adjustments.

It is thus not easy from the data to conclude about success factors in the programmes. Feedback from some supervisors, however, clearly indicates that funding of the student's research is crucial to the supervisor's interest in recruiting students to the programme. We found a remarkable difference in both level of and system of funding between the universities. It also seems clear that the four universities have quite different strategies when it comes to running own courses or not. NTNU and UiB have none or almost none own credits to offer their students, but rely on the ordinary PhD programme. Their students thus have to take courses together with ordinary PhD students, who are both older and more experienced. UiO and UiT offer their students 60–75% of all credits needed within the programme. There are both pros and cons to these two strategies, and it is interesting that the programmes seem divided in these respects. Students from NTNU and UiB had lower satisfaction with the services of the local Programme. Whether this relative dissatisfaction is partly associated with less funding and fewer courses from the Programme is not possible to elucidate, but seems plausible.

### An inspiration in training of medical doctors

The Medical Student Research Programme also has effects beyond the specific goal of increased recruitment to research. It can be supposed that the programmes promote the students' interest in research-based medicine generally, as well as providing individual students with the ability to delve deeper into a specific field. Students learn how to use methodological tools and gain increased knowledge about the choice of methods, data collection and data analysis. The training should develop the individual student's critical thinking and interpretation of data and literature. Furthermore the students learn to communicate their own research findings. In this regard, most of the Programme students felt that it was also useful for standard medical studies. The Medical Student Research Programme gives research experience, and also focus on research in the whole student environment. The Research Programme students have themselves established an annual conference, Frampeik, for all medical students with an interest in research [9]. Graduating from the two-year Medical Student Research Programme will contribute to increased level of competence in medicine also for the students who do not go on to do a PhD. The Research Programme is now publicly acknowledged in that graduation from the Programme reduces the specialist training time by one year in all specialist fields. When the Research Programme was introduced, some people feared there would be a split in medical training, i.e. into researchers and physicians [10]. Given the faculties' intentions [11], such worries appear to be unfounded. Nor is there any basis for claiming that the Medical Student Research Programme has become an "elite establishment" for medical students who think they are better than others. On the contrary, the Medical Student Research Programmes appear to stimulate an increased interest in research in a broad range of students, and also to make the research role among medical doctors more commonplace.

### Medical Student Research Programmes and the Quality Reform

The Quality Reform of higher education came into force in 2002, as Norway's answer to the European Bologna Process [12,13]. With the exception of the structure and titles of qualifications, the Quality Reform applies to the training of medical doctors, and thus also to the Medical Student Research Programme, which means that all study quality requirements and system requirements must be met. The Quality Reform is well implemented in several areas in the Medical Student Research Programmes, particularly when it comes to structuring, study progress and quality control. Documentation of supervision and individual follow-up are much more rigorous than for ordinary subjects and topics in medical school. A binding contract of supervision is agreed, and personal supervision is given with feedback on presentations at seminars, courses, etc., as well as close follow-up from the faculties. However, as far as the Quality Reform's qualification system is concerned, the Research Programme model is a little more problematic. Students on the Research Programmes have not obtained a higher degree, and can thus formally not participate in a PhD-programme, something which is presupposed by the scheme. In the same way, it is assumed that scientific results in the shape of a research project and scientific publications should be able to be included in any future PhD. These are fundamental conditions of the Medical Student Research Programme, part of its rationale and existence, which is not in line with the strict three-part system of qualification in the Quality Reform (bachelor, master, PhD).

## Conclusion

The Norwegian Medical Student Research Programme has been successful in fulfilling the goal of increased participation in medical research by medical students and graduated physicians. The students are well distributed between the sexes and are choosing a broad spectrum of research fields. They write scientific articles, give specialist presentations at national and international conferences, and make international contacts through field studies abroad. Most of the students have ambitions of doing a PhD, i.e. they want to continue their research after having completed the Research Programme. The Medical Student Research Programmes have had a good start, but it is still too early to judge the final results, i.e. whether it will lead to more medical doctors with a PhD, more and better publications and a strengthening of Norwegian medicine internationally. Financial and organisational continuity are therefore essential in order that the Research Programme model may be tested in its entirety.

## Abbreviations

UiB: (University of Bergen); UiO: (University of Oslo); NTNU: (Norwegian University of Science and Technology); UiT: (University of Tromsø).

## Competing interests

The authors declare that they have no competing interests.

## Authors' contributions

All authors designed and planned the study. KF and JBH performed the initial analyses. JBH drafted the first report in Norwegian, all authors contributed to the manuscript. SH drafted the present paper, all authors contributed to the manuscript. All authors have approved the final manuscript.

## Pre-publication history

The pre-publication history for this paper can be accessed here:


